# The Effect of Ultrasound on the Rehydration Characteristics of Semi-Dried Salted *Apostichopus japonicus*

**DOI:** 10.3390/foods12244382

**Published:** 2023-12-05

**Authors:** Xiaoyan Wang, Yongchang Su, Yangduo Wang, Xiaoting Chen, Xiaoe Chen, Zhiyu Liu

**Affiliations:** 1 College of Food and Pharmacy, Zhejiang Ocean University, Zhoushan 316022, China; 2 Key Laboratory of Cultivation and High-value Utilization of Marine Organisms in Fujian Province, Fisheries Research Institute of Fujian, National Research and Development Center for Marine Fish Processing (Xiamen), Xiamen 361013, China; suyongchang5@126.com (Y.S.);

**Keywords:** semi-dry salted *Apostichopus japonicus*, ultrasound, rehydration, low-field nuclear magnetic resonance, response surface method, scanning electron microscope

## Abstract

To effectively shorten the rehydration time of *Apostichopus japonicus* and reduce the nutrient loss during the rehydration process, an ultrasound-assisted rehydration method was adopted to rehydrate semi-dry salted *A. japonicus* in this study. The effects of different ultrasonic powers, temperatures, and times on the rehydration characteristics, textural characteristics, and sensory quality of the semi-dry salted *A. japonicus* were studied. Box–Behnken response surface analysis was used to study the influence of the interactions among the three factors on the rehydration ratio of the semi-dry salted *A. japonicus*, and a quadratic multinomic regression model was established to predict the optimal rehydration ratio. The results showed that ultrasound could change the structure of semi-dry salted *A. japonicus* and form a spatial network structure, thereby improving its water absorption capacity and reducing rehydration time. The optimal rehydration effect could be obtained when the ultrasonic power was 400 W, the ultrasonic temperature was 50 °C, and the ultrasonic time was 83 min. Ultrasonic power, ultrasonic time, and ultrasonic temperature influenced the rehydration ratio of the semi-dry salted *A. japonicus*. Under the optimal rehydration conditions in this study, the rehydration ratio of semi-dry salted *A. japonicus* obtained by the test was 2.103, which was consistent with the value predicted by the Box–Behnken response surface method.

## 1. Introduction

As a typical marine organism in the family of vertebrates and echinodermata [1,2], sea cucumbers are rich in mucopolysaccharides [3], amino acids [4], fatty acids [5], collagen [6], and saponins [7]. Sea cucumber has attracted considerable attention because of its nutritional value [8,9]. However, fresh sea cucumbers are prone to autolysis and spoilage in certain environments, because they contain a large number of proteins and autolytic enzymes [10]. Therefore, once sea cucumbers leave an appropriate growth environment, it is not conducive to their survival. Therefore, there are considerable challenges associated with the storage and transportation of sea cucumbers. To solve the problems caused by the self-dissolution of sea cucumbers, more than 80% of fresh sea cucumbers that are captured worldwide are dried, such as light-dried sea cucumbers, salt-dried sea cucumbers, freeze-dried sea cucumbers, and salted sea cucumbers, for storage and sale [11]. Semi-dried salted *Apostichopus japonicus* is a semi-wet and semi-dried *A. japonicus* made from fresh and dried by salting the plants in a salt tank several times. High-quality salted sea cucumbers, pickled salted sea cucumbers, and semi-dry salted sea cucumbers are the main salted sea cucumbers that are available on the market. A large amount of salt is added to salted sea cucumbers during processing to slow the reproductive ability of microorganisms, thus greatly improving the storage time of sea cucumbers [12]. Low-temperature cooking and drying are used to process semi-dry salted sea cucumbers to retain the body integrity and nutrition of fresh sea cucumbers to the greatest extent. Moreover, because part of the water in the semi-dried salted *A. japonicus* is removed during processing, it is easier to store and keep fresh than fresh *A. japonicus*. However, semi-dry salted *A. japonicus* still needs to be cooked and soaked repeatedly before consumption, which causes a loss of water-soluble nutrients. Therefore, it is necessary to adopt an efficient rehydration method for semi-dry salted *A. japonicus*.

Most studies on the rehydration characteristics of sea cucumbers have focused on dried sea cucumbers [13,14,15]. Fukunaga et al. [16] studied the rehydration characteristics of dried sea cucumbers in different fluids, such as pure water, K_2_CO_3_ solution, crude tea water, and rice washing water. The results showed that the moisture content of dried sea cucumbers was the highest after a full soaking in pure water, and the rehydration rate was the fastest in the K_2_CO_3_ solution. The hardness of the sea cucumbers soaked in crude tea water was the highest, whereas that of the sea cucumbers soaked in rice washing water was the lowest. Nuclear magnetic resonance (NMR) H-1 T-2 was used by Geng et al. to monitor the water absorption and distribution during rehydration of lightly dried sea cucumbers [13]. Their results showed that the optimum prepreg time for dried sea cucumbers was 24 h, and the optimum rehydration time was 96 h. Texture profile analysis (TPA) and Masson staining were used by Xu et al. to study the effects of rehydration conditions on the microstructure and textural properties of dried sea cucumbers [17]. The results showed that the cooking temperature has a significant influence on the hardness, adhesion, elasticity, cohesiveness, and resilience of sea cucumbers. To sum up, it is not difficult to conclude that there are few studies on the rehydration characteristics of semi-dry salted *A. Japonicus*. The existing rehydration methods, such as pure water, semi-oil, steam, and ultra-high-pressure rehydration, have many disadvantages, including nutrient loss, long rehydration times, and the poor taste of sea cucumbers after rehydration. Because ultrasonic treatment can significantly improve the mass transfer characteristics of the material without increasing the temperature of the material, the rehydration of dry food by ultrasonic treatment may have broad application prospects.

Based on the hypothesis that the rehydration effect of food can be enhanced by ultrasonic treatment, some scholars have used ultrasonic-assisted technology to study the rehydration characteristics of some foods, such as brown rice and chickpeas. Lu et al. [18] observed the microstructure of brown rice after ultrasonic treatment. They found that the microstructure of rice was changed by ultrasound, which made it easier for water to enter the brown rice, thus accelerating the water absorption rate. Wambura et al. compared the use of ultrasound to rehydrate rice with the traditional pure water soaking method [19]. The results showed that the rehydration time of the ultrasound-assisted method was reduced by 70% compared with that of the traditional method. The influence of ultrasound with different frequencies and powers on the water absorption rate of chickpeas during soaking was compared by Yildirim et al. [20,21]. The results showed that ultrasound with a low frequency and high power could improve the water diffusion rate in chickpeas. At present, Huang has studied the changes in the main nutrient contents of sea cucumbers treated by a traditional rehydration method and an ultrasonic-assisted rehydration method before and after rehydration [22]. The results show that the water content, protein, fat, and total sugar of sea cucumbers after ultrasonic-assisted rehydration are higher than those after traditional rehydration. Thus, it is feasible to apply ultrasound for the rehydration of semi-dry salted *A. japonicus*. The strength of the ultrasonic effect is related to the ultrasonic frequency, power, and treatment time, which are the main factors affecting the rehydration characteristics of sea cucumbers.

Based on the disadvantages of existing rehydration methods and the feasibility of ultrasonic-assisted rehydration methods, to effectively shorten the rehydration time of semi-dry salted *A. japonicus* and reduce nutrient loss during the rehydration process, an ultrasound-assisted rehydration method was adopted. The rehydration times of semi-dry salted *A. japonicus* treated with ultrasound and the traditional soaking method were compared. The effects of different ultrasonic powers, temperatures, and times on the rehydration characteristics, textural characteristics, and sensory quality of semi-dry salted *A. japonicus* were studied. Moreover, low-field nuclear magnetic resonance and scanning electron microscopy were used to observe the water migration in semi-dry salted *A. japonicus* during rehydration and its microstructure after ultrasonic treatment. This study provides a theoretical basis for the processing, storage, and consumption of semi-dry salted *A. japonicus* and other dried foods.

## 2. Materials and Methods

### 2.1. Materials and Instruments

The semi-dried salted *A. japonicus* were purchased from Ningde, Fujian, China, with an individual weight of 4.5 ± 0.2 g. Ultrasonic instrument (KQ-500DE) was obtained from Kunshan ultrasonic instrument Co., Ltd. (Kunshan, Jiangsu, China). A texture analyzer (TA-XTplus) was obtained from Stable Microsystems (Godalming, UK). The electronic balance (BS124S) was purchased from Sartorius GMBH (Goettingen, Germany). The nuclear magnetic resonance analysis and imaging system (MesoMR) was obtained from Shanghai Niumai Electronic Technology Co., Ltd. (Shanghai, China). A vacuum-sealing machine (YS-ZS-420XL) was obtained from Quanzhou Jukun Machinery Co., Ltd. (Quanzhou, Fujian, China). Carbon dioxide critical point dryer (XD-1) was obtained from Eiko, Ltd. (Osaka, Japan). The ion-gilding apparatus (IB-3) was obtained from Eiko Ltd. (Japan). A scanning electron microscope (SEM; JSM-840) was obtained from JEOL Ltd. (Tokyo, Japan).

### 2.2. Pretreatment of Raw Materials

**Figure 1:**
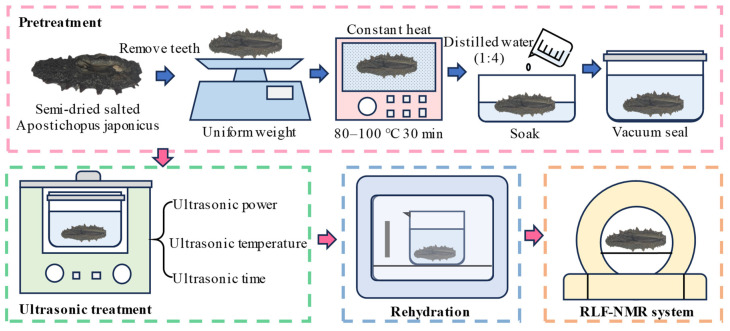
Experimental procedure.

The pretreatment method for the raw materials that was adopted in this study was modified from previous studies [16,23]. The experimental procedure is illustrated in Figure 1. First, a pair of scissors was used to cut the abdomen of *A. japonicus* to remove the calcareous matter and teeth from the mouth. After cleaning, each *A. japonicus* sample was weighed accurately (0.001 g). Then, the semi-dry salted *A. japonicus* were added to distilled water heated to 80–100 °C. Sea cucumbers were heated at a constant temperature for 30 min, removed, and cooled. Finally, the treated sea cucumbers were added to distilled water at a mass ratio of 1:4 and vacuum sealed in a polyethylene vacuum plastic bag for subsequent ultrasonic treatment. There are two reasons for soaking the test material. Firstly, the semi-dry salted *A. japonicus* was sealed in vacuum after adding water, which can improve the transmission effect of ultrasound in the semi-dry salted *A. japonicus*. Secondly, the rehydration process can be promoted by the vibration and the rupture of cavitation bubbles that are caused by ultrasound.

### 2.3. Traditional Rehydration Methods

Six pretreated semi-dry salted *A. japonicus* samples were placed in 500 mL beakers. Distilled water was then added to the beaker, and the mass ratio of material to liquid was 1:4. Finally, the beaker was sealed with plastic wrap and placed in a refrigerator at 4 °C, and the water was changed every 12 h.

### 2.4. Ultrasound-Assisted Rehydration

The ultrasonic treatment system used in this study is an indirect system, where cavitation is conveyed through a water bath. First, the semi-dried salted *A. japonicus,* sealed in a polyethylene vacuum plastic bag, is put into the ultrasonic instrument. And then, water is added into the tank until the water submerges the semi-dried salted *A. japonicus* sample. Finally, the sample is treated using ultrasound.

#### 2.4.1. Ultrasound Power

Because different ultrasonic powers can produce different vibration intensities and cavitation effects, the influence of ultrasonic power on the rehydration characteristics of the semi-dry salted *A. japonicus* was mainly analyzed in this study. The ultrasonic frequency, ultrasonic time, and ultrasonic temperature are set to constants of 45 kHz, 60 min, and 40 °C, respectively, while the ultrasonic power is a variable with a range of 200–400 W. The ultrasonic power of 200–400 W used in this study can be obtained by directly adjusting the ultrasonic instrument. The semi-dry salted *A. japonicus,* treated using ultrasound, were placed in clean distilled water (mass ratio of 1:4) in a refrigerator at 0–4 °C. Water was changed every 12 h, and after soaking for 24 h, the sea cucumbers were removed, drained, and weighed. The optimal ultrasonic power was determined by the degree to which semi-dry salted *A. japonicus* returned to their original fresh state after rehydration and by textural characteristics including hardness, elasticity, mastication, and cohesiveness, as well as sensory characteristics including color, chewability, umami, odor, and morphology.

#### 2.4.2. Ultrasonic Temperature

The ultrasonic frequency, ultrasonic time, and ultrasonic power are set to constants of 45 kHz, 60 min, and 300 W, respectively, while the ultrasonic temperature is a variable with a range of 20–60 °C. When the temperature exceeds the set value, a certain volume of hot water in the instrument was discharged, and then, the same volume of flowing cold water was immediately added to the instrument. In this way, we can effectively ensure that the temperature is maintained at the set value. The semi-dry salted *A. japonicus,* treated using ultrasound, were placed in clean distilled water (mass ratio of 1:4) in a refrigerator at 0–4 °C. Water was changed every 12 h, and after soaking for 24 h, the sea cucumbers were removed, drained, and weighed. The optimal ultrasonic temperature was determined by measuring the rehydration ratio, texture, and sensory properties.

#### 2.4.3. Ultrasonic Time

The ultrasonic frequency, ultrasonic temperature, and ultrasonic power are set to constants of 45 kHz, 40 °C, and 300 W, respectively, while the ultrasonic temperature is a variable with a range of 20–100 min. The semi-dry salted *A. japonicus,* treated using ultrasound, were placed in clean distilled water (mass ratio of 1:4) in a refrigerator at 0–4 °C. Water was changed every 12 h, and after soaking for 24 h, the sea cucumbers were removed, drained, and weighed. The optimal ultrasonic treatment time was determined by measuring rehydration ratio, texture, and sensory properties.

### 2.5. Test of Response Surface Optimization Test

The optimal design variables can be obtained using the response surface method. Therefore, based on the influence of different ultrasonic conditions on the rehydration ratio of semi-dry salted *A. japonicus* and the Box–Behnken experimental design principle, three factors—ultrasonic power, temperature, and time—were selected as research objects. Three levels of each of these three factors are labeled as −1, 0, and 1. Response surface software Design Expert 10.0.3 was used to carry out co-design of factor levels, and then, the conditions for the optimal rehydration ratio were obtained. The factor levels are listed in Table 1.

#### 2.5.1. Measurement of the Rehydration Ratio

The rehydration ratio of semi-dry salted *A. japonicus* was used as an evaluation index of the rehydration effect. First, the pretreated semi-dry salted *A. japonicus* was weighed using an analytical balance. The excess water on the surface of the semi-dry salted *A. japonicus* after ultrasound-assisted rehydration was removed with absorbent paper, and the sea cucumber was weighed. The rehydration ratio (*R*_r_) is the mass ratio of sea cucumber before and after rehydration:

(1) $$R_{r}=m_{r}/m_{0}$$

where *m*_0_ and *m*_r_ are the masses of the semi-dry salted *A. japonicus* before and after rehydration, respectively. In this study, each group of samples was 6 semi-dry salted *A. japonicus,* and each sea cucumber was measured separately.

#### 2.5.2. Measurement of Textural Properties

After rehydration, the semi-dry salted *A. japonicus* were cut into small pieces measuring 2 × 2 × 1 cm^3^. The hardness, elasticity, mastication, cohesiveness, and textural characteristics of the back wall of the sea cucumber in the same part were determined in all experiments. A P/5 probe (a cylindrical stainless steel probe with a diameter of 5 mm) was selected for the measurement. The propulsion speed of the probe was always 1.0 mm/s, the trigger force was 5 g, and the degree of compression was 30%. The interval between two compressions was 5 s. The data acquisition rate was 200 ps.

#### 2.5.3. Sensory Evaluation

For the selection of evaluators, first of all, the potential evaluators were trained professionally to make them understand the principles, methods, and standards of the sensory evaluation. Then, the sensory ability, consistency, and reliability of evaluators were evaluated to ensure that they have the basic requirements for sensory evaluation. Finally, 20 people with sensory evaluation experience were selected through the above methods. The color, chewability, odor, umami, and morphology of the samples were evaluated in the same temperature and light space according to the sensory evaluation table (Table 2). Three parallel tests were performed for each sample and repeated three times. Informed consent was obtained from all subjects involved in the study.

#### 2.5.4. NMR Analysis Method

Water migration in semi-dry salted *A. japonicus* was measured using low-field nuclear magnetic resonance. After rehydration, the surface water of the semi-dry salt-cured specimens was drained and placed at the center of the permanent magnetic field. A 40 mm diameter RF probe coil was used for the measurement. The CPMG sequence parameters of multipulse echo are as follows: SW = 125 kHz, RFD = 0.08 ms, PRG = 1, TW = 4000 ms, NS = 8, TE = 0.2 ms, and NECH = 5000.

#### 2.5.5. NRI Analysis Method

Magnetic resonance imaging (MRI) data were acquired using a 40 mm diameter RF probe coil. The experimental parameters of the low-field MRI were as follows: TR = 500 ms and TE = 18.125 ms. The mapped image was processed with pseudo-color.

#### 2.5.6. Scanning Electron Microscopy (SEM)

(1) Sampling and fixation: Body wall samples of semi-dry salted *A. japonicus* were collected and fixed in a 2% glutaraldehyde fixation solution. (2) Cleaning: A total of 0.1 MPBS buffer was used to clean the semi-dry salted *A. japonicus* three times for 10 min each. (3) Dehydration: The semi-dry salted *A. japonicus* was successively dehydrated with 30%, 50%, 70%, 80%, and 100% alcohol, including 100% ethanol, twice, for 10 min each. (4) A CO_2_ critical point dryer was used to dry semi-dry salted *A. japonicus*. (5) The surface of semi-dry salted *A. japonicus* was coated with a gold spray. (6) Semi-dry salted *A. japonicus* were observed using scanning electron microscopy.

#### 2.5.7. Data Processing

Each experiment was conducted in parallel 3 times, and the data were expressed as mean ± standard deviation. Microsoft Office Excel 2016 was used for data statistics, SPSS 17.0 was used for difference significance analysis, Origin 2018 was used for plotting. Design-Expert software (version 13.0) was used to design the response surface test and construct the model under different ultrasonic conditions. *p* < 0.05 indicated a significant difference; *p* < 0.01 meant an extremely significant difference.

## 3. Results and Discussion

### 3.1. Analysis of Ultrasound-Assisted Rehydration Methods

#### 3.1.1. Effect of Ultrasonic Power on the Rehydration Characteristics

**Figure 2:**
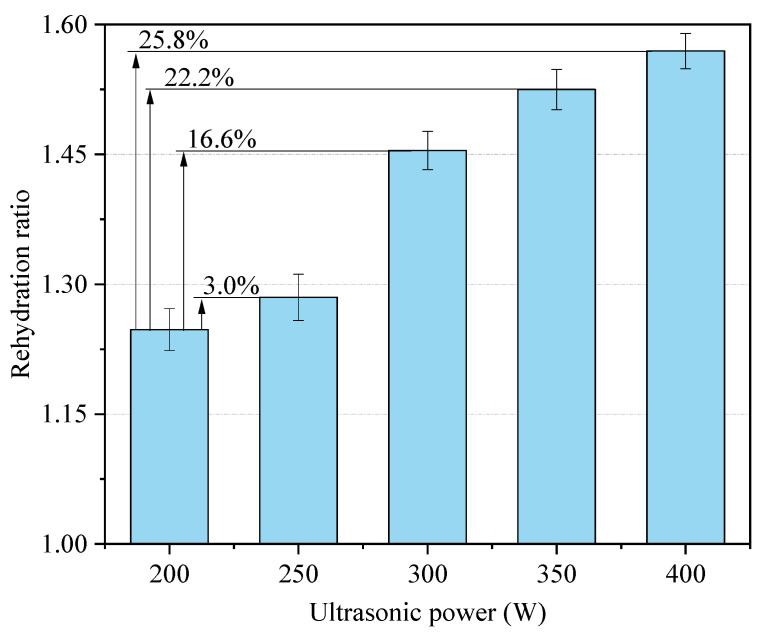
Effect of ultrasonic power on the rehydration ratio.

In this study, ultrasonic powers of 200, 250, 300, 350, and 400 W were used to conduct ultrasound-assisted rehydration treatment on semi-dry salted *A. japonicus*, and the rehydration ratio after 24 h is shown in Figure 2. When the ultrasonic power was 200–300 W, the rehydration ratio of the semi-dry salted *A. japonicus* showed a trend of a slow increase at first, followed by a sharp increase with increasing ultrasonic power. However, when the ultrasonic power was 300–400 W, the rehydration rate increased slowly. The maximum rehydration rate of the semi-dry salted *A. japonicus* at 400 W was 1.57 (*p* < 0.05), which was 25.8% higher than that at 200 W. This is because the high ultrasonic power causes the ultrasound to generate extremely high temperatures and pressures in water, that is, the cavitation effect of ultrasound. This effect results in tiny eddies and water flow in the water, which accelerates the diffusion and absorption of water within the semi-dry salted *A. japonicus*, thereby accelerating the rehydration rate. In summary, the best rehydration effect was obtained by controlling the ultrasonic power to 300–400 W within the parameters adopted in this study.

**Figure 3:**
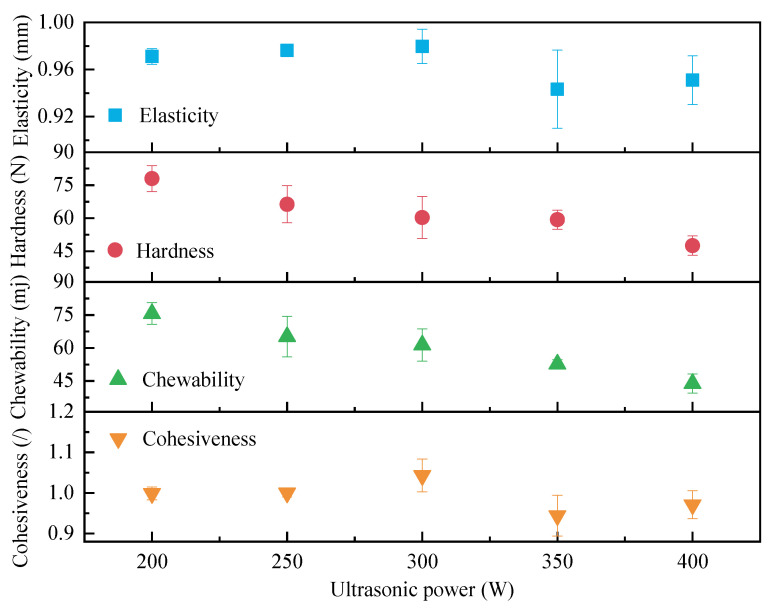
Effect of ultrasonic power on textural characteristics.

Textural characteristics are an important index for evaluating the quality of semi-dry salted *A. japonicus* following rehydration. In this study, semi-dry salted *A. japonicus* was treated with ultrasound at ultrasonic powers of 200, 250, 300, 350, 400, 450, and 500 W, and the results of the texture determination after rehydration for 24 h are shown in Figure 3. The hardness, elasticity, mastication, and cohesiveness of the semi-dry salted *A. japonicum* that were treated with different ultrasonic powers were significantly different. With an increase in ultrasonic power, the hardness and chewability of semi-dry salted *A. japonicus* decreased, whereas the elasticity and cohesiveness increased and then decreased. Hardness reflects the force required for semi-dry salted *A. japonicus* to reach a given deformation range, and an increase in moisture content can lead to a decrease in hardness [24]. This was mainly due to the cavitation effect caused by the ultrasound. The tiny bubble vibration in the water and the change in the water flow cause vibration, collision, and friction of the tiny structures of molecules and cells in the semi-dry salted *A. japonicus*. This leads to the denaturation of nutrients, proteins, and polysaccharides, resulting in changes in hardness, elasticity, chewability, and cohesiveness.

**Figure 4:**
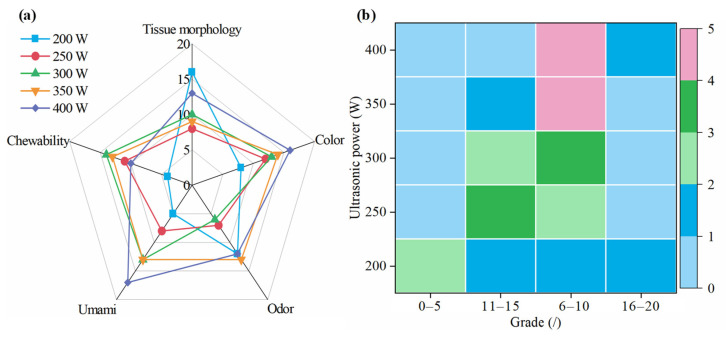
Sensory evaluation of semi-dry salted *Apostichopus japonicus* at different ultrasonic powers. (**a**) Score for each item and (**b**) distribution of scores.

Semi-dry salted *A. japonicus* treated with ultrasound at different ultrasonic powers was used for sensory evaluation after rehydration for 24 h, and the results of the sensory evaluation are shown in Figure 4. The color, chewability, odor, umami taste, and histological morphology of the semi-dry salted *A. japonicus* after ultrasonic treatment at different ultrasonic powers were obviously different. When the ultrasonic power is 400 W, 300 W, 350 W, 400 W, and 200 W, the best color, chewability, odor, umami, and morphology of the semi-dry salted A. japonicum can be obtained, respectively. The total score of each sensory evaluation from 200 W to 400 W was 68, 62, 56, 46, and 45, respectively; that is, the total scores were ordered as 400 W > 350 W > 300 W > 200 W > 250 W. Compared with the semi-dry salted *A. japonicus* treated using ultrasound with an ultrasonic power of 200–350 W, the color, umami, and total score of the *A. japonicus* treated with an ultrasonic power of 400 W were the highest, at 16, 17, and 64, respectively. Therefore, semi-dry salted *A. japonicus* obtained the best sensory evaluation when the ultrasonic power was controlled at 300–400 W.

#### 3.1.2. Effect of Ultrasonic Temperature on the Rehydration Characteristics

**Figure 5:**
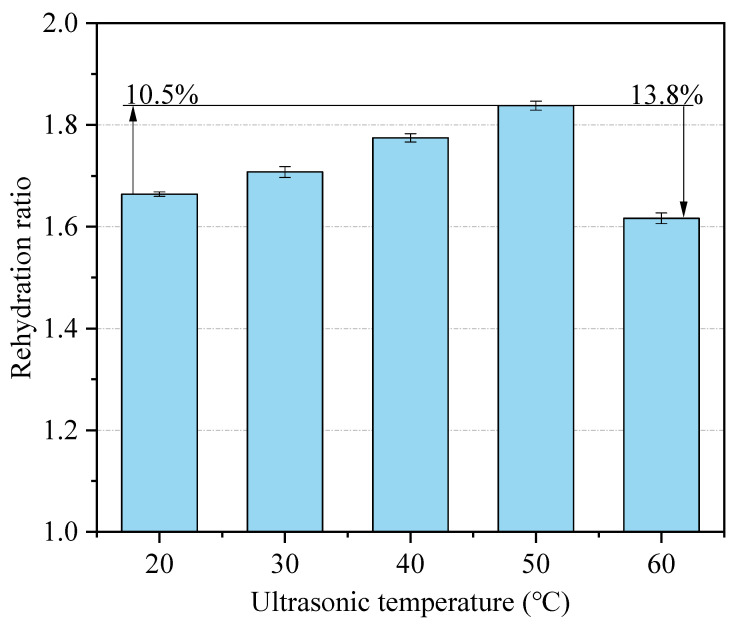
Effect of ultrasonic temperature on rehydration ratio.

In this study, the semi-dry salted *A. japonicus* are treated using ultrasound with ultrasonic temperatures of 20 °C, 30 °C, 40 °C, and 50 °C, respectively, and the results for the rehydration ratio after 24 h are shown in Figure 5. When the ultrasonic temperature is between 20 °C and 50 °C, the rehydration ratio of the *A. japonicus* increases with the increase in the temperature. The maximum rehydration ratio of the semi-dry salted *A. japonicus* is 1.84 (*p* < 0.05) at 50 °C, which is 10.5% higher than that at 20 °C. However, when the ultrasonic temperature increases from 50 °C to 60 °C, the rehydration ratio of the *A. japonicus* decreases. This is because the body wall of the semi-dry salted *A. japonicus* could stretch out with an increase in the ultrasonic temperature within a certain temperature range. The collagen fibers in the body wall loosen, which promotes the dissolution of salt from the body and the integration of water from the outside. Therefore, when the ultrasonic temperature is lower than 50 °C, the rehydration ratio of the semi-dry salted *A. japonicus* can be increased by increasing the ultrasonic temperature. However, when the ultrasonic temperature exceeds 50 °C, the protein denaturation occurs in the semi-dry salted *A. japonicus*, which weakens the water retention capacity of the *A. japonicus*. Therefore, the rehydration ratio of the semi-dry salted *A. japonicus* decreases sharply when the temperature is higher than 50 °C. Therefore, the best rehydration effect could be obtained by controlling the ultrasonic temperature to 30–50 °C within the parameters adopted in this study.

**Figure 6:**
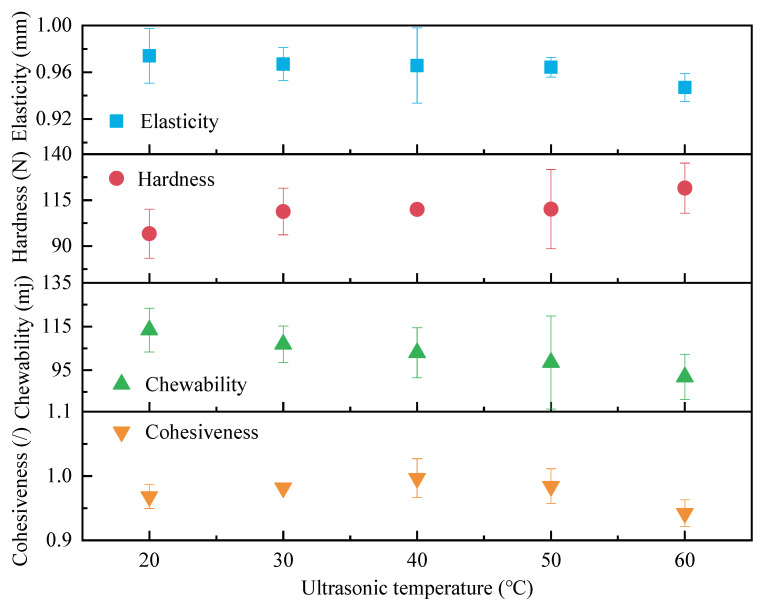
Effect of ultrasonic temperature on textural characteristics.

Semi-dry salted *A. japonicus* treated with ultrasound at different temperatures was used for texture determination after rehydration for 24 h, and the results of the texture determination are shown in Figure 6. With an increase in ultrasonic temperature, the hardness of the semi-dry salted *A. japonicus* gradually increased, cohesiveness first increased and then decreased, and elasticity and mastication gradually decreased. This is because the collagen fibers in the body of semi-dry salted *A. japonicus* gradually shrank with an increasing temperature. This causes the body wall of *A. japonicus* to shrink, and its hardness gradually increases. The body wall of the semi-dry salted *A. japonicus* gradually softened with an increase in the ultrasonic temperature. As a result, the elasticity of collagen fibers in the body wall of *A. japonicus* was reduced. The chewability of semi-dry salted *A. japonicus* depends greatly on its hardness and elasticity; therefore, the chewability of *A. japonicus* decreases with an increase in temperature. On the other hand, when the ultrasonic temperature is higher than 40 °C, the polysaccharide, protein, and other molecules in the semi-dry salted *A. japonicus* change, which reduces its internal stability and cohesion.

**Figure 7:**
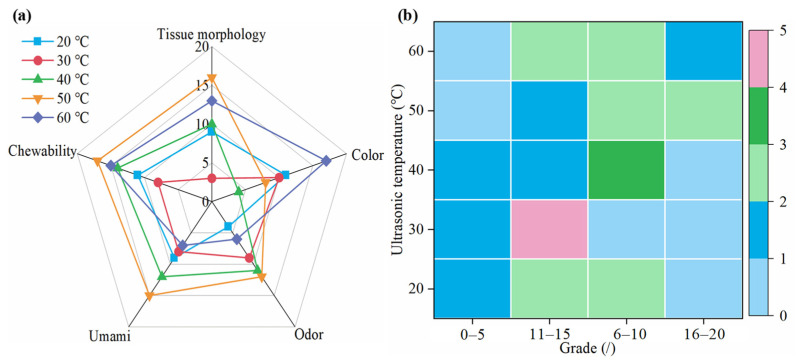
Sensory evaluation of semi-dry salted *Apostichopus japonicus* at different ultrasonic temperatures. (**a**) Score for each item and (**b**) distribution of scores.

The sensory evaluation results for semi-dry salted *A. japonicus* treated with ultrasound at different ultrasonic temperatures at the same rehydration time are shown in Figure 7. The color, chewability, odor, umami taste, and histological morphology of the semi-dry salted *A. japonicus* after ultrasonic treatment at different temperatures were obviously different. When the ultrasonic temperature is 60 °C, 50 °C, 50 °C, 50 °C, and 50 °C, the best color, chewability, odor, umami, and morphology of the semi-dry salted A. japonicum can be obtained, respectively. The total score of each sensory evaluation from 20 °C to 60 °C is 68, 58, 51, 44, and 38, respectively, that is, the total scores are ordered as 50 °C > 60 °C > 40 °C > 20 °C > 30 °C. Compared with the semi-dry salted *A. japonicus* treated using ultrasound with other ultrasonic temperatures, the morphology, odor, umami, chewability, and total score of the *A. japonicus* treated with an ultrasonic temperature of 50 °C are the highest. Therefore, the semi-dry salted *A. japonicus* can obtain the best sensory evaluation when the ultrasonic temperature is controlled at 40–60 °C.

#### 3.1.3. Effect of Ultrasonic Treatment Time on Rehydration Characteristics

**Figure 8:**
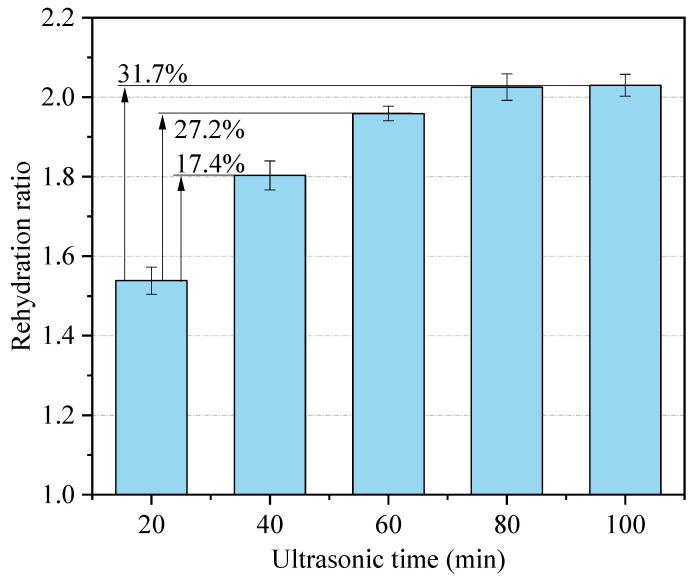
Effect of ultrasonic treatment time on the rehydration ratio.

The semi-dry salted *A. japonicus* was treated using ultrasound for 20, 40, 60, 80, and 100 min, and the results of the rehydration ratio are shown in Figure 8. As shown in Figure 8, the rehydration ratio of the semi-dry salted *A. japonicus* increased with an increasing ultrasonication time. The maximum rehydration ratio of the semi-dry salted *A. japonicus* was 2.07 (*p* < 0.05) at an ultrasonic treatment time of 100 min, which was 31.7% higher than that at 20 min. When the ultrasonic treatment time was longer than 80 min, the rehydration ratio of *A. japonicus* gradually increased. The cavitation effect caused by ultrasound releases energy and increases the internal temperature of the semi-dry salted *A. japonicus*, which helps water penetrate into the interior of the cells of the semi-dry salted *A. japonicus*. Longer rehydration times can cause *A. japonicus* to absorb more water and improve the rehydration ratio. However, the texture and natural morphology of the semi-dry salted *A. japonicus* would change if the ultrasonic time is too long, which would affect the rehydration ratio of *A. japonicus*. In summary, it is best to control the ultrasonic treatment time to 60–100 min.

**Figure 9:**
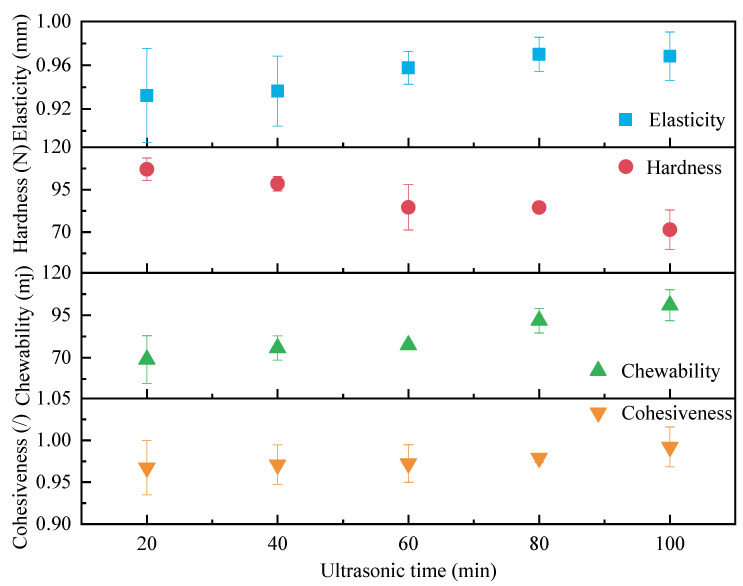
Effect of ultrasonic treatment time on textural characteristics.

Semi-dry salted *A. japonicus* treated using ultrasound for different durations was used for texture determination after rehydration for 24 h, and the results of the texture determination are shown in Figure 9. With an increase in the ultrasonic treatment time, the hardness of the semi-dry salted *A. japonicus* after rehydration showed a decreasing trend, whereas the elasticity, mastication, and cohesiveness showed an increasing trend and tended to be stable. The moisture content of the semi-dry salted *A. japonicus* increased, and the hardness decreased with increasing ultrasonication time. Owing to the continuous diffusion of water molecules into the interior of the semi-dry salted *A. japonicus*, the elasticity of collagen fibrin in the body wall of *A. japonicus* increases as a result of water absorption and swelling. When the ultrasonication time exceeded 60 min, this effect gradually decreased; therefore, the elasticity initially increased and then flattened. The structures of macromolecules, such as proteins and polysaccharides, in the semi-dry salted *A. japonicus* changed with an increase in the ultrasonic treatment time, and the texture of the body wall of the semi-dry salted *A. japonicus* became dense and firm. Therefore, the mastication and cohesiveness of semi-dry salted *A. japonicus* increased after rehydration.

**Figure 10:**
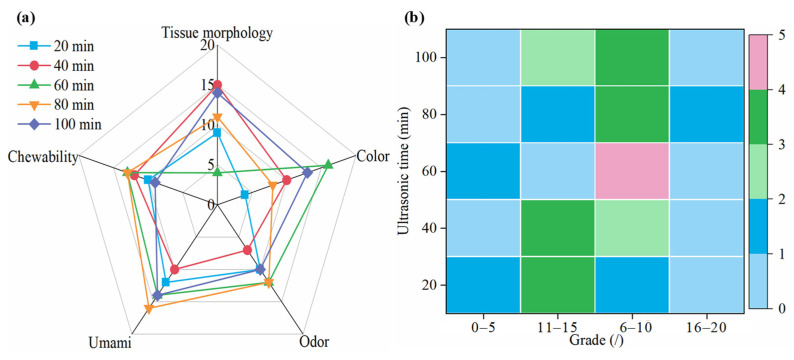
Sensory evaluation of semi-dry salted *Apostichopus japonicus* at different ultrasonic treatment times. (**a**) Score for each item and (**b**) distribution of scores.

The sensory evaluation results for the semi-dry salted *A. japonicus* treated with ultrasound for different durations at the same rehydration time are shown in Figure 10. The color, chewability, odor, umami taste, and histological morphology of the semi-dry salted *A. japonicus* after ultrasonic treatment differed at different ultrasonic times. When the ultrasonication time is 60 min, 60 min, 60 min, 80 min, and 40 min, the best color, chewability, odor, umami, and morphology of the semi-dry salted A. japonicum can be obtained, respectively. The total scores of each sensory evaluation from 20 min to 100 min were 60, 60, 59, 54, and 45, respectively; that is, the total scores were ordered as follows: 80 min = 100 min > 60 min > 40 min > 20 min. Compared with the semi-dry salted *A. japonicus* treated with ultrasound for 20–80 min, the morphology, color, and total score of *A. japonicus* treated with ultrasound for 100 min were the highest. Therefore, semi-dry salted *A. japonicus* obtained the best sensory evaluation when the ultrasonic treatment time was controlled at 60–100 min.

### 3.2. Design and Results of the Response Surface

Using the data processing software Design Expert 10.0.3, data related to each factor were input into the Box–Behnken data analysis software (Design Expert 13), and the design table of the response surface test was obtained. The experimental data were then obtained according to the factor levels in the design table, as listed in Table 3.

#### 3.2.1. Establishment and Analysis of Rehydration Ratio Model

Based on the results in Table 3, the data analysis software Design expert 10.0.3 is used to perform multiple regression fitting on the test data. The ultrasonic power, ultrasonic temperature, and ultrasonic time were named as A, B, and C, respectively, and the rehydration ratio was used as the response value for multiple regression fitting. The coefficient and significance test results of the regression model are shown in Table 4. The quadratic multinomial regression model is obtained as follows:

(2) $$\begin{array}{c} Y=2.06+0.1\;\times A-0.036\;\times B+0.093\;\times C+0.00015\;\times \mathrm{AB}\;-\;0.071\;\times \mathrm{AC}+0.056\;\times \mathrm{BC}\\ -0.076\;\times A^{2}\;-\;0.15\;\times B^{2}\;-\;0.11\;\times C^{2} \end{array}$$

Based on the analysis results of the rehydration ratio model and regression coefficients in Table 4, it can be seen that *p* < 0.001 (extremely significant) and its lack of fit *p* = 0.2541 > 0.05 (insignificant) to the regression model indicate a good degree of fit. Thus, the corresponding values of the regression equations were predicted. In addition, the regression coefficient R^2^ of the model was 0.9793, and the adjusted R^2^ was 0.9526 (>0.8000). This indicates that 95.26% of the data can be explained by this model, indicating that Equation (2) has a high reliability.

The F-value is an important index for evaluating the impact of each factor on a response. The larger the F-value, the higher the contribution of the model component to the response is. When the probability of significance was *p* < 0.05, the factor had a significant effect on the response value. As shown in Table 4, the ultrasonic time and power significantly affected the rehydration ratio (*p* < 0.01), and the ultrasonic temperature also had a significant effect on the rehydration ratio (*p* < 0.05). Therefore, the main effect relationship of each factor was A > C > B, that is, ultrasonic power > ultrasonic time > ultrasonic temperature. The quadratic interaction of AC had a significant effect on the rehydration ratio (*p* < 0.01), BC had a significant effect on the rehydration ratio (*p* < 0.05), and AB had an insignificant effect on the rehydration ratio (*p* > 0.05). Therefore, the order of the degree of influence of secondary interactions on the rehydration ratio was AC > BC > AB.

#### 3.2.2. Interaction among Various Factors

**Figure 11:**
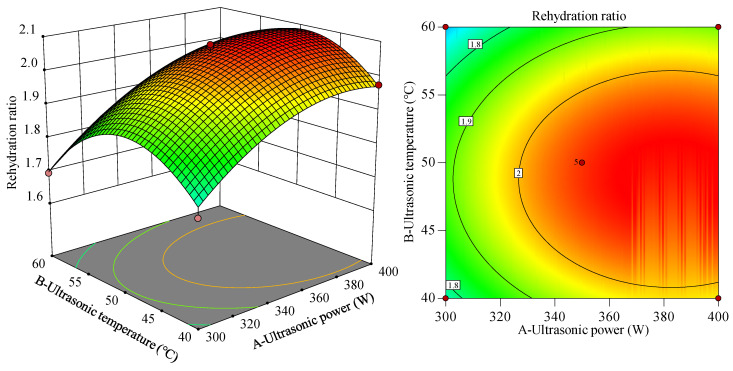
Response surface diagram and contour plot of the ultrasonic power and ultrasonic temperature to the rehydration ratio. In response surface diagram, the change of response value can be represented by color gradient or color block. Generally, red represents a higher response value while green represents a lower response value.

According to the regression equation, the influence of the ultrasonic power, ultrasonic temperature, and ultrasonic time on the rehydration ratio was analyzed based on the shape of the response surface and the contour plot. The influence of these two factors on the response value can be determined by observing the steepness of the response surface slope. The steeper the response surface is, the more obvious the interaction between the two factors is. The effects of the ultrasonic power (A) and ultrasonic temperature (B) on the rehydration ratio are shown in Figure 11. In response surface diagram, the change of response value can be represented by color gradient or color block. Generally, red represents a higher response value while green represents a lower response value. As shown in Figure 11, on the AB interaction surface, the gradient of the rehydration ratio first increased and then flattened with an increase in ultrasonic power, whereas the gradient of the rehydration ratio first increased and then decreased with an increase in ultrasonic temperature. When the ultrasonic power is 340–400 W and the ultrasonic temperature is 45–55 °C, a large rehydration ratio can be obtained. In addition, the results obtained from Figure 11 are consistent with those of the variance analysis in Table 4.

**Figure 12:**
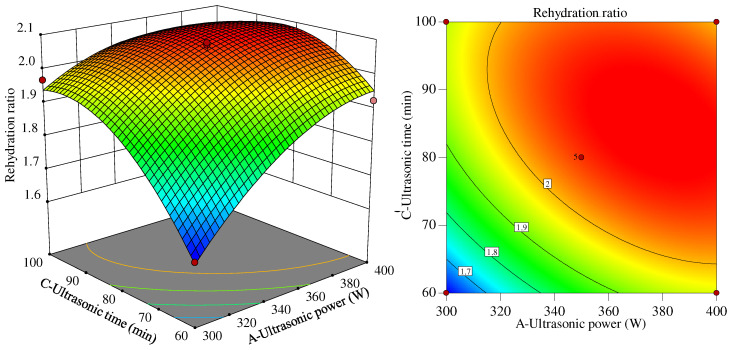
Response surface diagram and contour plot of the ultrasonic power and ultrasonic time to the rehydration ratio.

The effects of the ultrasonic power and time on the rehydration ratio are shown in Figure 12. The AC interaction surface in Figure 12 shows that the gradient of the rehydration ratio gradually increased with the increasing ultrasonic power. When the ultrasonic power was small, the gradient of the rehydration ratio first increased and then flattened with an increase in the ultrasonic time, whereas the gradient of the rehydration ratio first increased and then decreased with an increase in the ultrasonic time when the ultrasonic power was large. Therefore, there was a significant interaction between the ultrasonic power and the ultrasonic time. A high rehydration ratio was obtained when the ultrasonic power was 340–400 W, and when the ultrasonic time was approximately 80–100 min, a large rehydration ratio could be obtained.

**Figure 13:**
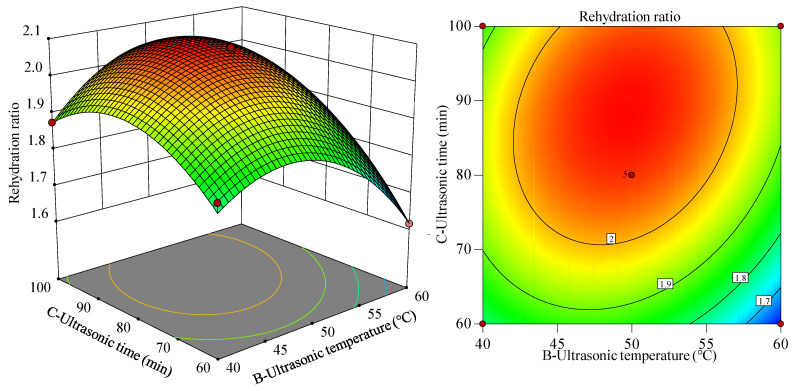
Response surface diagram and contour plots of ultrasonic temperature and ultrasonic time to the rehydration ratio.

The effects of the ultrasonic temperature and ultrasonic time on the rehydration ratio are shown in Figure 13. As shown in Figure 13, it can be seen from the BC interaction surface that with an increase in the ultrasonic time, the rehydration ratio shows a trend of first increasing and then flattening. When the ultrasonic time was short, the gradient of the rehydration ratio first increased and then decreased with an increasing ultrasonic temperature, whereas when the ultrasonic time was long, the gradient of the rehydration ratio first increased and then decreased with an increasing ultrasonic time. This indicates that the ultrasonic temperature and time have a strong interaction effect. When the ultrasonic temperature is 45–55 °C and the ultrasonic time is about 80–100 min, the rehydration ratio is large, and the results obtained from Figure 13 are also consistent with the results of the variance analysis in Table 4.

#### 3.2.3. Verification of Test Results

According to the regression equation, taking the maximum rehydration ratio as the optimization objective, the optimal conditions obtained by the prediction are an ultrasonic power of 379.512 W, ultrasonic temperature of 49.149 °C, and ultrasonic time of 83.159 min. According to the actual experimental conditions, the conditions are revised as ultrasonic power of 400 W, ultrasonic temperature of 50 °C, and ultrasonic time of 83 min. Under the optimal rehydration conditions, the actual rehydration ratio is 2.103 ± 0.0021 after three repeated experiments, which is within 5% of the predicted rehydration ratio of 2.10. Therefore, the values predicted in this study are consistent with the experimental values, indicating that the optimal processing technology obtained by the response surface method is reasonable.

### 3.3. Comparison of Ultrasound-Assisted and Traditional Soaking Methods

**Figure 14:**
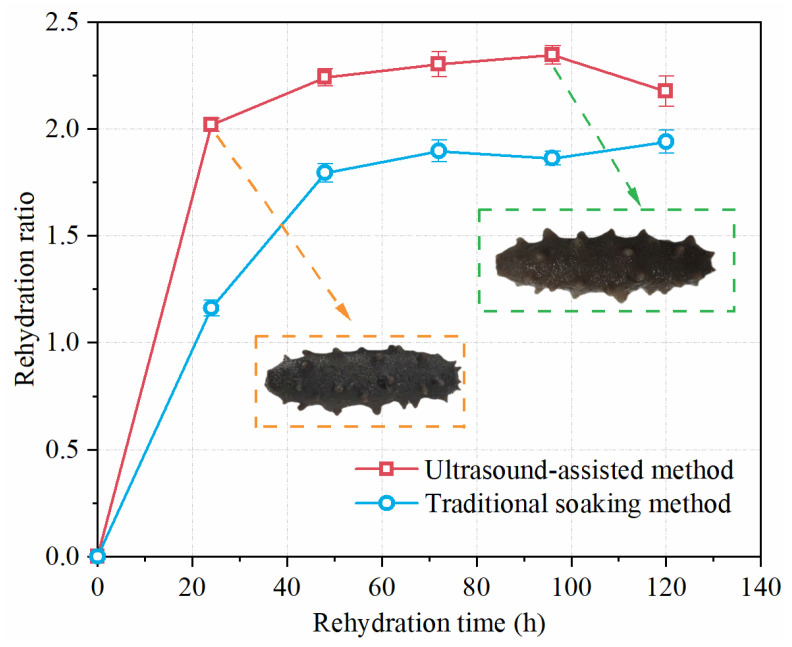
Effects of ultrasonic treatment and traditional soaking treatment on the rehydration ratio of semi-dry salted *Apostichopus japonicus.* The arrows and dashed box show the photos of the rehydrated *A. japonicus*.

Semi-dry salted *A. japonicus* was rehydrated using ultrasonic-assisted and traditional soaking methods, and the rehydration ratios of the two methods at 24, 48, 72, 96, and 120 h were measured, as shown in Figure 14. The arrows and dashed boxes in Figure 14 show the photos of the rehydrated *A. japonicus*. The results show that the maximum rehydration ratio of the ultrasonic-assisted method is 2.35, which is 23.04% higher than that of the traditional soaking method (1.91). The rehydration ratio of the semi-dry salted *A. japonicus* treated with the ultrasound-assisted method increased rapidly from 0 to 48 h. Subsequently, the rehydration ratio increased slowly and approached the end of rehydration at 48–96 h. After 96 h, the rehydration rate decreased. This is because the rehydration process of semi-dry salted *A. japonicus* accelerated and decelerated rehydration. When sea cucumbers reach an equilibrium state, water absorption stops, and the rehydration ratio tends to be flat or starts to decline. For semi-dry salted *A. japonicus* treated using the traditional soaking method, the rehydration ratio also increased sharply within 0–48 h, but its growth rate was lower than that of the ultrasound-assisted method. After 96 h, the rehydration ratio continued to increase, and the sea cucumbers did not reach the end of rehydration. In general, the ultrasound-assisted method can shorten the rehydration time and increase the rehydration rate compared with the traditional soaking method.

### 3.4. NMR Analysis

**Figure 15:**
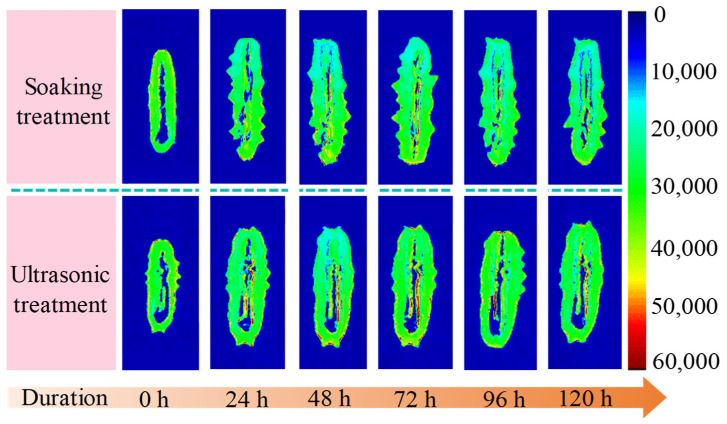
Water distribution in semi-dry salted *Apostichopus japonicus* treated with ultrasonic and traditional soaking methods.

As a fast, direct, accurate, and nondestructive food detection method [25,26,27], MRI can not only determine the water distribution in food, but also visually display the changes in the internal structure of food during processing. Accordingly, LF-NMR and LF-MRI are widely used in the water distribution monitoring of various foods such as seafood [28,29,30]. Figure 15 displays the proton density of the semi-dry salted *A. japonicus* at different rehydration times treated by ultrasonic and traditional soaking methods, which could show the water distribution and individual size differences of *A. japonicus*. In Figure 15, red indicates a strong signal intensity, and blue indicates a weak signal intensity; the higher the signal strength, the greater the moisture content. As shown in Figure 15, the volume of the semi-dry salted *A. japonicus* treated using the ultrasound-assisted method and the signal intensity of its central inner region gradually increased with an increasing rehydration time. The results showed that the moisture content of *A. japonicus* treated using the ultrasound-assisted method was high. In addition, the volume of the semi-dry salted *A. japonicus* treated with the ultrasound-assisted method increased faster than that of the semi-dry salted *A. japonicus* treated with the traditional soaking method. However, the signal intensity and volume growth rate of the *A. japonicus* treated with the traditional soaking method were low during the entire rehydration period. In conclusion, the ultrasonic treatment significantly enhanced the water absorption capacity of semi-dry salted *A. japonicus*.

### 3.5. MRI Analysis

**Figure 16:**
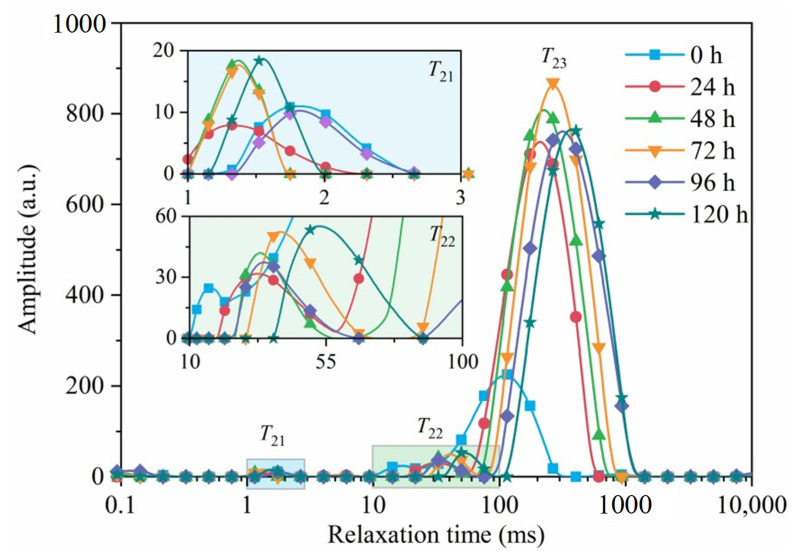
Transverse relaxation of semi-dry salted *Apostichopus japonicus* treated with ultrasonic and traditional soaking methods at different rehydration times.

Figure 16 shows the transverse relaxation of semi-dry salted *A. japonicus* treated with ultrasonic soaking under different rehydration times. In the figure, the semi-dry salted *A. japonicus* with different rehydration times produced three peaks: *T*_21_ (0.1–10 ms), *T*_22_ (10–100 ms), and *T*_23_ (100–1000 ms), which represent bound, immobile, and free water in the cells, respectively [31]. As shown in Figure 16, with an increasing rehydration time, *T*_21_, *T*_22_, and *T*_23_ gradually increased, and *T*_2_ changed toward a long relaxation trend. These results indicate that the water mobility of semi-dry salted *A. japonicus* can be enhanced by increasing the rehydration time. During the rehydration process of the semi-dry salted *A. japonicus*, water molecules gradually penetrated from the outside of the cells into the inside of the *A. japonicus*, resulting in a large increase in the free water content. This process involves a constant conversion between bound, immobile, and free water. This is because ultrasound could be used to accelerate the change in collagen structure through cavitation and mechanical effects, which further promotes the formation of a spatial network structure in *A. japonicus*. The increase in the number of micropores in the tissues was more conducive to free water entering the spatial network structure of the semi-dry salted *A. japonicus* collagen.

**Figure 17:**
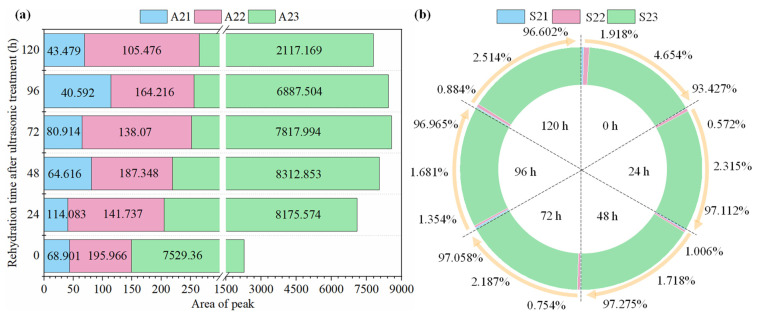
Peak area of semi-dry salted *Apostichopus japonicus* treated by the ultrasound-assisted method at different rehydration periods. (**a**) Peak area and (**b**) peak area proportion.

The relative contents (*A*_21_, *A*_22_, and *A*_23_) of water in the different phases (bound water, immovable water, and free water) of the semi-dry salted *A. japonicus* under different rehydration times are shown in Figure 17a. The larger the peak area, the higher the water content is in this phase. Figure 17b shows the total content (*S*_21_, *S*_22_, and *S*_23_) of water in different phases (bound water, immovable water, and free water) in semi-dry salted *A. japonicus*. The larger the peak ratio, the greater the proportion of water is in this phase. It can be seen from Figure 17a,b that both *A*_22_ (the content of immobile water) and *S*_22_ (the proportion of immobile water) of the rehydrated semi-dry salted *A. japonicus* from 24 to 120 h show a decreasing trend compared with that at 0 h. Between 0 and 72 h, the total water content of *A. japonicus* increased, and the free water content and proportion increased sharply from 0 to 48 h. This is because with an increase in the rehydration time, a certain network structure is formed in the semi-dry salted *A. japonicus*, which can promote the absorption of water by *A. japonicus*. Therefore, the free water content of the semi-dry salted *A. japonicus* increased. After 72 h, the total water content, free water content, and proportion of semi-dry salted *A. japonicus* decreased. This is because a long rehydration time can destroy the network structure of collagen fibers and reduce the water-holding capacity of *A. japonicus*. Therefore, the optimum rehydration time for the semi-dry salted *A. japonicus* treated using the ultrasound-assisted method was 72 h.

### 3.6. SEM Image Analysis

**Figure 18:**
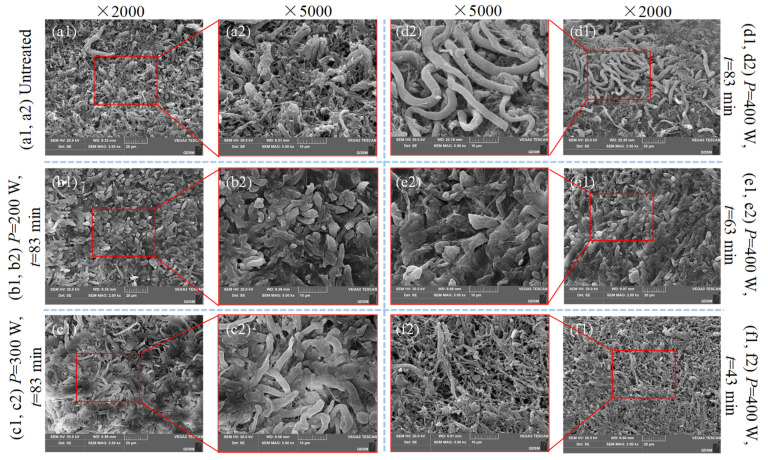
Microstructure of semi-dry salted *Apostichopus japonicus* subjected to different ultrasonic treatments.

Figure 18 shows the influence of different ultrasonic powers and times on the microstructure of semi-dry salted *A. japonicus*. The rehydration rate of semi-dry salted *A. japonicus* is closely related to the compactness of its structure. As shown in Figure 18a, the semi-dry salted *A. japonicus* without ultrasonic treatment exhibited a more compact structure and smaller structural gaps and holes. As can be seen from the comparison of Figure 18a–d, with an increase in ultrasonic power, the structural gap of the semi-dry salted *A. japonicus* gradually increased, the microstructure of the pores increased, the collagen structure became loose, and a complex spatial network structure was formed, which was more conducive to the entry of external water molecules into the tissue to improve the absorption capacity of the water molecules of the semi-dry salted *A. japonicus*. Compared with Figure 18a,d–f, it can be seen that with the extension of the ultrasonic treatment time, the collagen fiber structure of the semi-dry salted *A. japonicus* also became loose and porous, forming a three-dimensional spatial network structure, exposing more hydrophilic groups, and increasing the adsorption capacity of free water, constantly absorbing water and expanding rapidly. This is consistent with the conclusion that ultrasonic treatment increases the rehydration ratio, shortens the rehydration time, and improves the textural characteristics of semi-dry salted *A. japonicus*.

Although the ultrasonic-assisted rehydration method can shorten the rehydration time, reduce the loss of nutrients, improve production efficiency, and reduce the time and cost of manual operation, it is still facing challenges in industrial applications such as energy consumption, large-scale production, and market demand. Firstly, ultrasound equipment needs to consume energy to generate high-frequency vibration. In industrial-scale applications, the energy consumption and cost-effectiveness of ultrasound equipment need to be considered. Secondly, the application of ultrasonic-assisted rehydration technology in industrial production needs to consider the problem of large-scale production, which includes equipment design and process automation. Thirdly, the industrial application of ultrasonic-assisted rehydration technology also needs to consider the market demand and consumer acceptance.

## 4. Conclusions

In this study, an ultrasound-assisted rehydration method was adopted to rehydrate semi-dry salted *A. japonicus*. The effects of different ultrasonic powers, temperatures, and times on the rehydration characteristics, textural characteristics, and sensory quality of semi-dry salted *A. japonicus* were studied. The main conclusions of this study are as follows:

(a) Compared with the traditional soaking method, the rehydration time of semi-dry salted *A. japonicus* can be shortened to 72 h using the ultrasound-assisted method, and the rehydration rate can be improved.
(b) The cavitation effect produced by ultrasound causes a spatial network structure to form in the body of the semi-dry salted *A. japonicus*, which promotes water absorption and increases the free water content in its body. The water absorption of semi-dry salted *A. japonicus* was enhanced using an ultrasonic-assisted method.
(c) The optimal rehydration effect could be obtained when the ultrasonic power was 400 W, the ultrasonic temperature was 50 °C, and the ultrasonic time was 83 min. Ultrasonic power, ultrasonic time, and ultrasonic temperature influenced the rehydration ratio of semi-dry salted *A. japonicus*.
(d) Under the optimal rehydration conditions in this study, the rehydration ratio of semi-dry salted *A. japonicus* obtained by the tests was 2.103, which was within 5% of the predicted rehydration ratio of 2.10. The quadratic polynomial regression model established in this study for predicting the optimal rehydration ratio is reasonable and effective.

## Figures and Tables

**Table 1:** Experimental conditions for factor-level response surface analysis.

Factor	Level		
	−1	0	1
A—Ultrasonic power (W)	300	350	400
B—Ultrasonic temperature (°C)	40	50	60
C—Ultrasonic time (min)	60	80	100

**Table 2:** Sensory evaluation of semi-dry salted *Apostichopus japonicus* treated with ultrasound.

Item	Grade			
	1–5	6–10	11–15	16–20
Color	White, Nonuniform color	Light brown or light yellow, Nonuniform color	Light brown or tan, Slightly uniform color	Black brown or black gray, Uniform color
Chewability	Soft, Nonelastic	Slightly soft, Poor elasticity	Hard, Elastic	Good elasticity, Moderate hardness
Umami	No umami, Funny flavor	Slightly umami, Odorless	Strong umami	Rich umami
Odor	Scentless, Fishy	Faint scent, Slightly fishy	Obviously fragrant, Tasteless	Excellent fragrance, Tasteless
Morphology	Incompleteness, Soft thorn	Slightly complete, Slightly soft thorn	Complete, Slightly straight thorn	Complete, Straight thorn

**Table 3:** Experimental design and results of response surface method for optimizing the rehydration ratio.

Case	A—Ultrasonic Power (W)	B—Ultrasonic Temperature (°C)	C—Ultrasonic Time (min)	Rehydration Ratio
1	300	40	80	1.7404
2	400	40	80	1.9747
3	300	60	80	1.6935
4	400	60	80	1.9284
5	400	50	100	1.9919
6	300	50	60	1.6162
7	350	60	100	1.8884
8	300	50	100	1.968
9	350	40	100	1.8751
10	400	50	60	1.9241
11	350	40	60	1.825
12	350	50	80	2.0835
13	350	60	60	1.6155
14	350	50	80	2.0239
15	350	50	80	2.0835
16	350	50	80	2.0769
17	350	50	80	2.0372

**Table 4:** Analysis results of the rehydration ratio model and regression coefficients.

Item	Sum of Squares of Deviations	Degree of Freedom	Mean Square	F-Value	*p*-Value	Significance Level
Model	0.38	9	0.042	36.73	<0.0001	**
A—Ultrasonic power	0.08	1	0.08	69.65	<0.0001	**
B—Ultrasonic temperature	0.01	1	0.01	9.09	0.0195	*
C—Ultrasonic time	0.069	1	0.069	59.86	0.0001	**
AB	9.00 × 10^−8^	1	9.00 × 10^−8^	7.82 × 10^−5^	0.9932	
AC	0.02	1	0.02	17.51	0.0041	**
BC	0.012	1	0.012	10.78	0.0134	*
A^2^	0.025	1	0.025	21.32	0.0024	**
B^2^	0.095	1	0.095	82.71	<0.0001	**
C^2^	0.051	1	0.051	43.92	0.0003	**
Residual	8.06 × 10^−3^	7	1.15 × 10^−3^			
Lack of fit	4.85 × 10^−3^	3	1.62 × 10^−3^	2.02	0.2541	ns
Pure error	3.21 × 10^−3^	4	8.02 × 10^−4^			
Total	0.39	16				

Note: R^2^ = 0.9793, Adj R^2^ = 0.9526, Pre R^2^ = 0.7874; *p* < 0.01 is extremely significant (**), *p* < 0.05 is significant (*), *p* > 0.05 is insignificant (ns).

## Data Availability

Data are contained within the article.
